# MiR-452-5p promotes colorectal cancer progression by regulating an ERK/MAPK positive feedback loop

**DOI:** 10.18632/aging.202657

**Published:** 2021-03-03

**Authors:** Xin Lin, Lu Han, Chuncai Gu, Yihong Lai, Qiuhua Lai, Qingyuan Li, Chengcheng He, Yan Meng, Lei Pan, Side Liu, Aimin Li

**Affiliations:** 1 Guangdong Provincial Key Laboratory of Gastroenterology, Department of Gastroenterology, Nanfang Hospital, Southern Medical University, Guangzhou, Guangdong, People’s Republic of China

**Keywords:** miR-452-5p, colorectal cancer, ERK/MAPK, PKN2, DUSP6

## Abstract

Background: MiR-452-5p plays an essential role in the development of a variety of tumors, but little is known about its biological function and mechanism in colorectal cancer (CRC).

Methods: The expression levels of miR-452-5p in CRC tissues and cells were detected by real-time quantitative PCR (qRT-PCR). Besides, the biological effects of miR-452-5p on CRC were investigated by functional experiments *in vitro* and *in vivo*. Furthermore, bioinformatics analysis, dual-luciferase reporter assay, chromatin immunecipitation assay, western blotting and recovery experiments were implemented to investigate the underlying molecular mechanism.

Results: The expression level of miR-452-5p was up-regulated in CRC tissues. MiR-452-5p promoted CRC cell proliferation, cell cycle transition and chemoresistance, and inhibited cell apoptosis. Moreover, miR-452-5p directly targeted PKN2 and DUSP6 and subsequently activated the ERK/MAPK signaling pathway, and it was transcriptionally regulated by c-Jun.

Conclusion: To conclude, miR-452-5p expression is up-regulated in CRC, which promotes the progression of CRC by activating the miR-452-5p—PKN2/DUSP6—c-Jun positive feedback loop. These findings indicate that miR-452-5p may act as a potential therapeutic target and clinical response biomarker for CRC.

## INTRODUCTION

Colorectal cancer (CRC) is the third most common cancer and a leading cause of cancer-related death worldwide [1–3]. In recent years, great achievements have been made to detect CRC earlier, but most patients are diagnosed at the advanced stages with poor outcomes. Therefore, it is urgently needed to more intensively investigate the molecular mechanism of CRC, so as to identify the early diagnostic markers and potential therapeutic targets of CRC.

MicroRNA (miRNA) is a class of the short single-stranded non-coding RNA, which interacts with mRNA through complementary sequence pairing of target gene 3' untranslated region (3'-UTR) [4–7], resulting in the degradation of mRNA or translation failure [8]. Accumulating evidence has reported that miRNAs are involved in the diagnosis and prognosis of CRC. MiR-135b, miR-320e and miR-30b are identified as the up-regulated miRNAs in CRC [9], and miR-30b plays a significant role in the growth of CRC, implicating that miRNAs can serve as the potential therapeutic approach to treat CRC [10]. MiR-452-5p has been reported to exert dual roles of a potential tumor suppressor gene and a candidate oncogene in multiple human cancers, including renal cancer, osteosarcoma, breast cancer, non-small cell lung cancer, hepatocellular carcinoma, prostate cancer and colorectal cancer [11–15]. However, its further molecular functions and mechanisms in CRC are less defined.

In the current study, we confirmed that the expression of miR-452-5p was up-regulated in CRC. Moreover, the exogenous transfection of miR-452-5p promoted CRC cell growth, cell cycle transition and chemoresistance, and inhibited cell apoptosis. PKN2 (protein kinase N2), the Rho/Rac effector protein and the serine/threonine protein kinase related to PKC, participates in the invasion, migration and adhesion of cells, as well as in actin cytoskeleton assembly, certain signal transduction, and cell cycle control [16]. DUSP6, also known as the dual specificity phosphatase 6, belongs to dual specificity protein phosphatase subfamily. It negatively regulates the mitogen-activated protein (MAP) kinase superfamily through the dephosphorylation of phosphoserine/threonine and phosphotyrosine residues [17]. PKN2 and DUSP6 are both the direct targets of miR-452-5p in CRC. Our study demonstrated that c-Jun up-regulated the expression of miR-452-5p, which activated the ERK/MAPK signaling pathway by inhibiting the PKN2/DUSP6 axis to form a positive feedback loop in CRC.

## RESULTS

### MiR-452-5p is up-regulated in CRC

**Figure 1:**
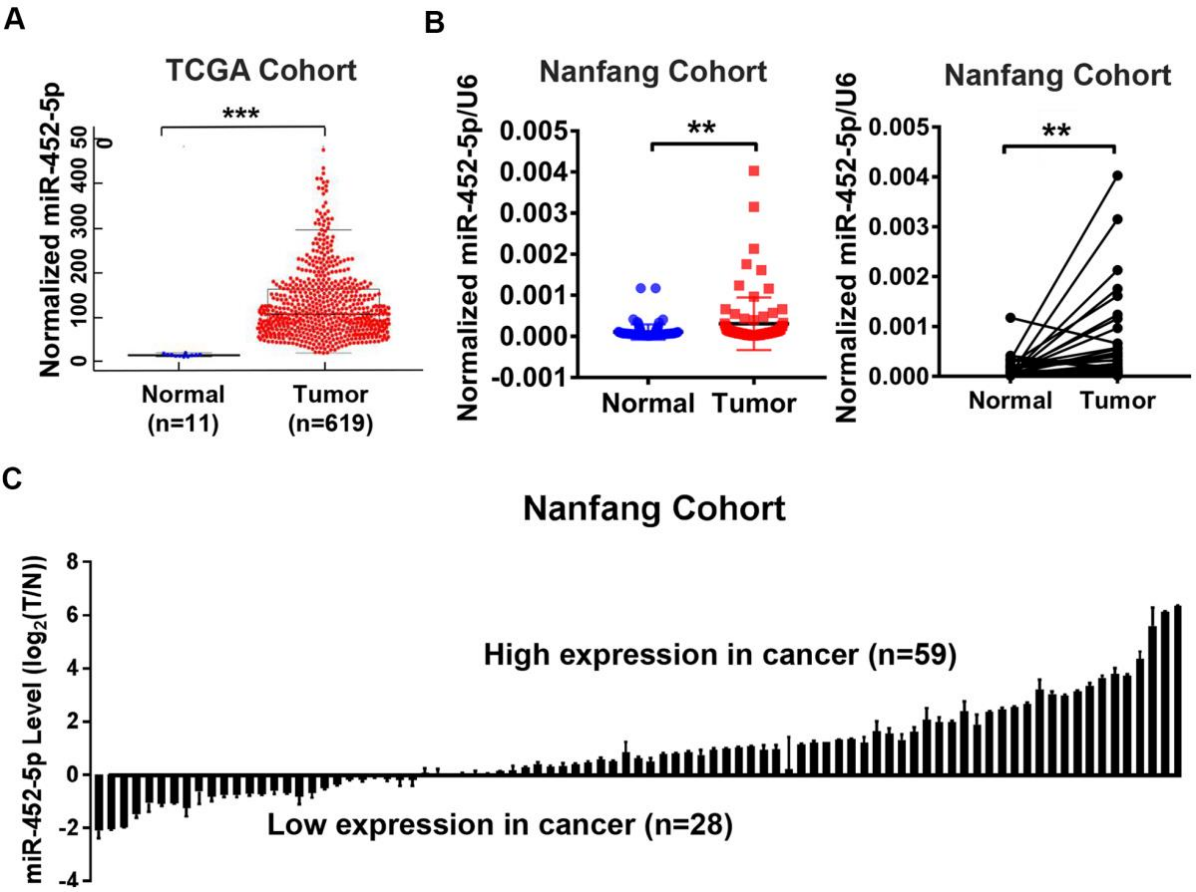
**The up-regulation of miR-452-5p in CRC.** (**A**) Bioinformatics analysis of miR-452-5p expression levels in colorectal cancer (T, n=619) and normal colorectal tissue (N, n=11) in TCGA database. (**B**) The expression levels of miR-452-5p in colorectal cancer tissues (T, n=87) and adjacent normal tissues (N, n=87) were detected by real-time quantitative PCR. (**C**) Waterfall plot showed the relative levels of miR-452-5p expression in colorectal cancer (T, n=87) and normal adjacent tissues (N, n=87).*p < 0.05, **p < 0.01, ***p < 0.001.

MiR-452-5p expression was analyzed using the Cancer Genome Atlas (TCGA) database, which indicated the up-regulation of miR-452-5p in CRC (Figure 1A). Furthermore, 87 CRC tissues and matched para-carcinoma mucosal tissue samples were used for real-time quantitative PCR (qRT-PCR) to verify the results. As a result, the miR-452-5p expression significantly increased in CRC tissues than in para-carcinoma mucosal tissue samples (Figure 1B, 1C). Analysis of clinicopathological features of 87 CRC patients showed that patients with high expression of miR-452-5p were worse in M stage and AJCC stage (Supplementary Table 1). These results indicate that miR-452-5p expression is up-regulated in CRC and associated with metastasis and poor prognosis.

### MiR-452-5p enhances cell proliferation of CRC both *in vivo* and *in vitro*

**Figure 2:**
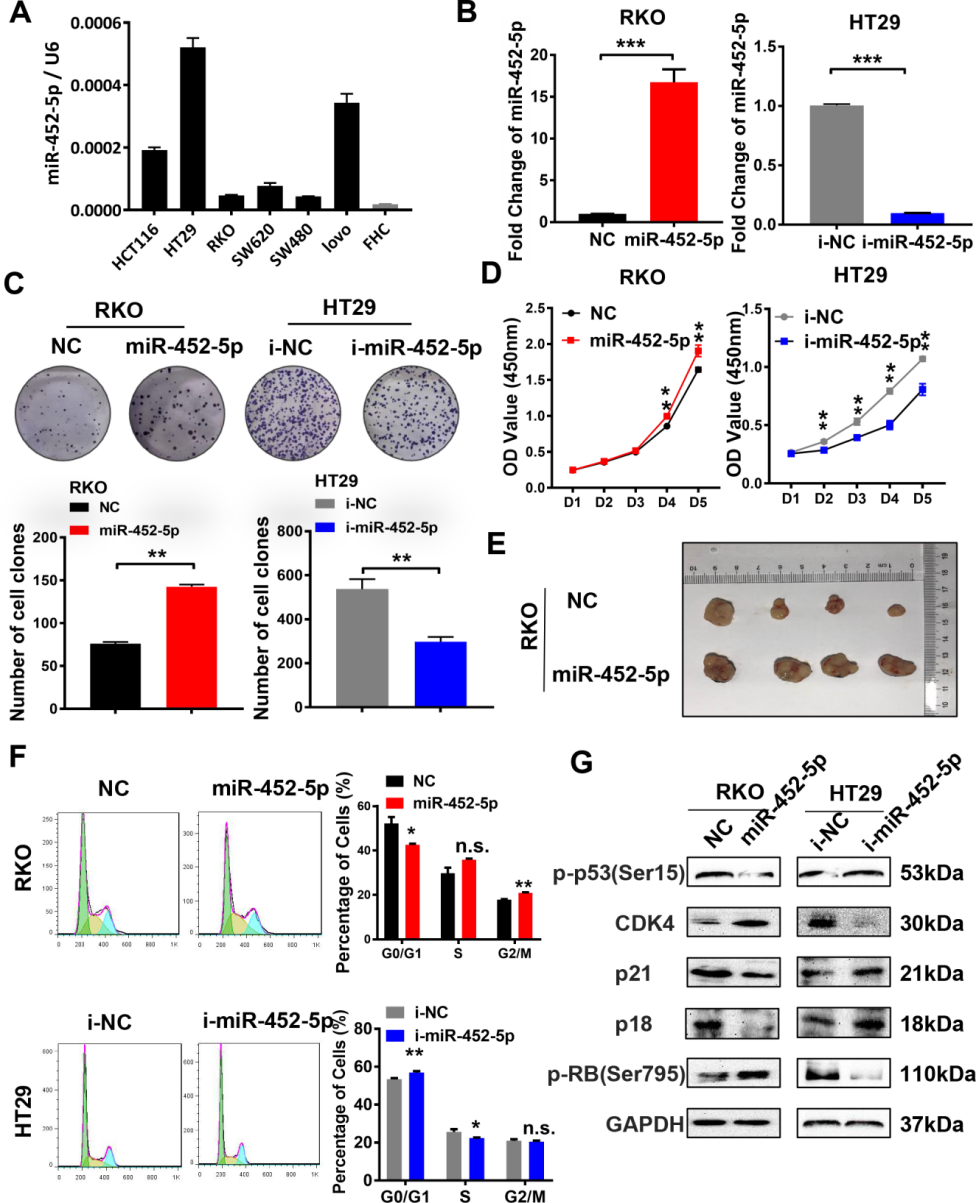
**MiR-452-5p promotes malignant biological behaviors of CRC cells.** (**A**) The expression levels of miR-452-5p in colorectal cancer cell lines and human normal intestinal epithelial cell line FHC. (**B**) Instantaneous transfection efficiency verification of miR-452-5p. (**C**) Effects of overexpression and knockdown of miR-452-5p on the proliferation of CRC cells: CCK8 assay. (**D**) Effects of overexpression and knockdown of miR-452-5p on the proliferation of CRC cells: panel cloning assay. (**E**) Effects of overexpression of miR-452-5p on subcutaneous tumor formation in nude mice. (**F**) Effects of overexpression and knockdown of miR-452-5p on CRC cell cycle: cell cycle detection by flow cytometry. (**G**) Cell cycle related-proteins detected by western blotting.*p < 0.05, **p < 0.01, ***p < 0.001, n.s. non-significant.

To further assess the biological functions of miR-452-5p in CRC, miR-452-5p expression was detected in normal colon epithelial cells (FHC) and six kinds of human CRC cells (LoVo, SW480, SW620, RKO, HT29, and HCT116) by qRT-PCR. According to our findings, miR-452-5p expression was relatively lower in the FHC cell line than in six kinds of CRC cells (Figure 2A). The RKO and HT29 cells with the least and highest miR-452-5p expression were chosen to transfect with miR-452-5p mimics and inhibitors, respectively. The transfection efficiency was confirmed by qRT-PCR (Figure 2B). To investigate the effect of miR-452-5p on CRC growth, CRC cell proliferation capacity *in vitro* was detected. As shown by clone formation assay, more clones were formed in RKO cells after miR-452-5p mimics transfection, while fewer clones were formed in HT29 cells transfected with miR-452-5p inhibitors (Figure 2C). Likewise, according to CCK-8 assay, miR-452-5p mimics transfection in RKO cells led to accelerated cell growth from day 4, while miR-452-5p inhibitor transfection in HT29 cells resulted in slower cell growth from day 2 (Figure 2D). Then, RKO-LV-NC and RKO-LV-miR-452-5p cells were inoculated subcutaneously to the nude mice, respectively, to determine whether miR-452-5p affected CRC proliferation *in vivo*. As a result, RKO-LV-miR-452-5p group displayed greater tumor size (Figure 2E), higher proliferation rate and heavier tumor weight (Supplementary Figure 1A, 1B). Moreover, to confirm the correlation of the proliferation promoting effect with cell cycle, cell cycle transformation was determined by flow cytometry. We revealed that miR-452-5p promoted the transformation of cell cycle in RKO cells, while miR-452-5p inhibitors in HT29 cells caused the arrest of cell cycle (Figure 2F). Also, western blotting was conducted to detect the cell cycle-associated protein levels. The results revealed that, miR-452-5p mimics down-regulated the protein levels of p53, p21 and p18 in RKO cells, while up-regulated those of CDK4 and p-rb. Inversely, miR-452-5p inhibitors up-regulated the expression of p18, p21 and p53 in HT29 cells, but down-regulated that of CDK4 and p-rb (Figure 2G and Supplementary Figure 3A). Taken together, miR-452-5p promoted the growth of CRC *in vitro* and *in vivo*, which indicated that miR-452-5p contributed to CRC growth.

### MiR-452-5p enhances resistance to 5-FU and reduces CRC apoptosis

**Figure 3:**
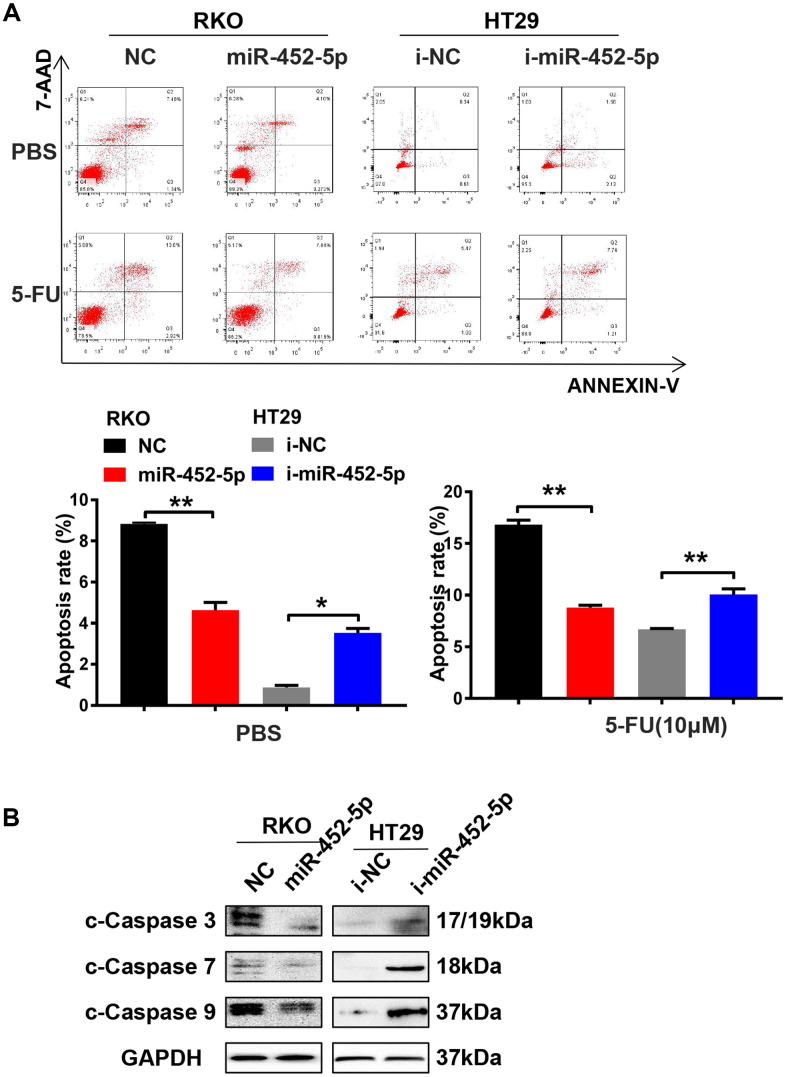
**MiR-452-5p enhances resistance to 5-FU and reduces CRC apoptosis.** (**A**) Detection of apoptosis in cells transfected with miR-452-5p and miR-452-5p inhibitor together with negative controls. (**B**) The expression of apoptosis-related proteins detected by western blotting.*p < 0.05, **p < 0.01, ***p < 0.001.

To further determine whether miR-452-5p affected CRC apoptosis, flow cytometry was conducted to investigate cell apoptosis. The results suggested that, overexpression of miR-452-5p reduced the apoptosis rate of RKO cells treated with PBS or 5-fluorouracil. Furthermore, HT29 cells treated with miR-452-5p inhibitors showed a higher apoptosis rate (Figure 3A). It suggested that miR-452-5p inhibited the chemosensitivity of CRC to 5-fluorouracil by reducing apoptosis. HT29 cells transfected with miR-452-5p inhibitors showed increased sensitivity to 5-FU and the IC50 values decreased, while the opposite results were observed in RKO cells with over-expression of miR-452-5p (Supplementary Figure 1C). Furthermore, the expression of apoptosis-associated proteins was detected by western blotting, which revealed that miR-452-5p mimics transfection in RKO cells led to down-regulation of apoptosis-associated protein levels, such as cleaved-caspase (c-caspase)3, c-caspase7, and c-caspase9. By contrast, transfection with miR-452-5p inhibitors up-regulated the levels of the above proteins within HT29 cells (Figure 3B and Supplementary Figure 3B). However, the full length caspases has also been tested but they do not show significant changes as the cleaved ones (Supplementary Figure 2A). These results indicated that, miR-452-5p repressed the apoptosis of cleaved-caspase proteins and thus reduced the chemosensitivity of CRC.

### MiR-452-5p directly targets PKN2 and DUSP6 in CRC cells

**Figure 4:**
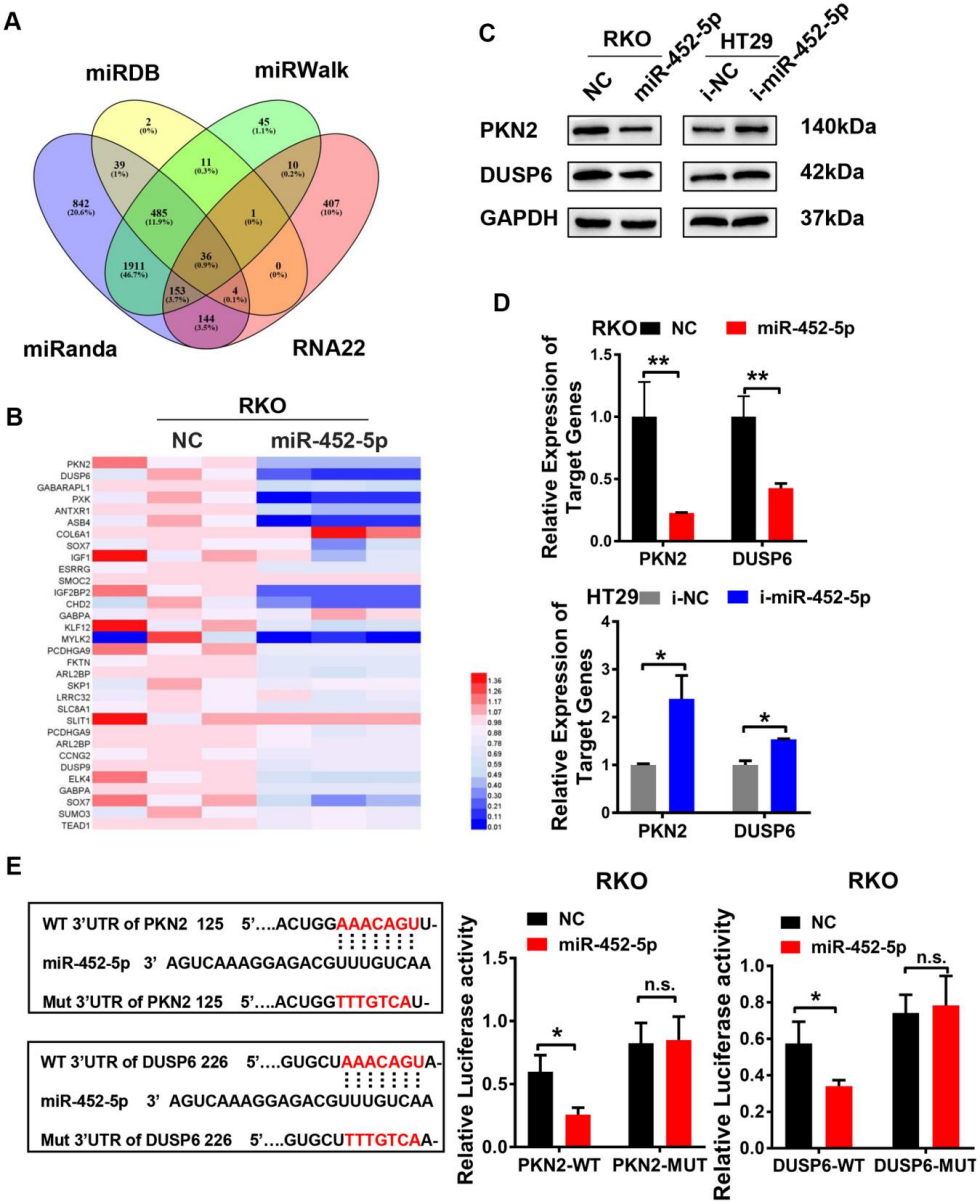
**MiR-452-5p directly targets PKN2 and DUSP6.** (**A**) Bioinformatics prediction of potential miR-452-5p target genes by 4 miRNA databases. (**B**) QRT-PCR results of candidate target genes. (**C**) The expression of PKN2 and DUSP6 after overexpression and knockdown of miR-452-5p detected by western blotting. (**D**) The expression of PKN2 and DUSP6 after overexpression and knockdown of miR-452-5p detected by qRT-PCR. (**E**) Luciferase reporter gene assay: binding sites and mutation sites of PKN2 and DUSP6 were shown on the left, luciferase reporter gene results were shown on the right. *p < 0.05, **p < 0.01, ***p < 0.001, n.s. non-significant.

Four public datasets (MiRDB, miRanda, miRwalk, and rna22) were used to figure out the miR-452-5p target genes. Finally, 36 candidate target genes were obtained (Figure 4A). Based on the interactions and the RNA sequencing results, a series of genes were selected to verify whether their expression was regulated by miR-452-5p. Then, we focused on PKN2 and DUSP6 (Figure 4B). Besides, the negative correlations of miR-452-5p with PKN2 and DUSP6 were further examined by western blotting and qRT-PCR assays (Figure 4C, 4D and Supplementary Figure 3C). To find out the candidate miR-452-5p binding regions in targeting PKN2 and DUSP6, the computer-based sequence analysis (MiRanda) was performed. The candidate binding regions in the 3′UTR of PKN2 and DUSP6 are shown in Figure 4E. To confirm whether PKN2 and DUSP6 were the miR-452-5p direct target genes, we generated 3′UTR regions in PKN2 and DUSP6 (PKN2-WT, DUSP6-WT), together with the matched mutants (PKN2-MUT, DUSP6-MUT). Afterwards, dual-luciferase reporter assay was conducted. It was shown that the activities of firefly luciferase in both 3′UTRs of PKN2-WT and DUSP6-WT were inhibited after co-transfection with miR-452-5p mimics, thus validating that PKN2 and DUSP6 were the direct target genes of miR-452-5p (Figure 4E).

### MiR-452-5p restrains the ERK/MAPK signaling pathway via suppression of PKN2/DUPS6 axis

**Figure 5:**
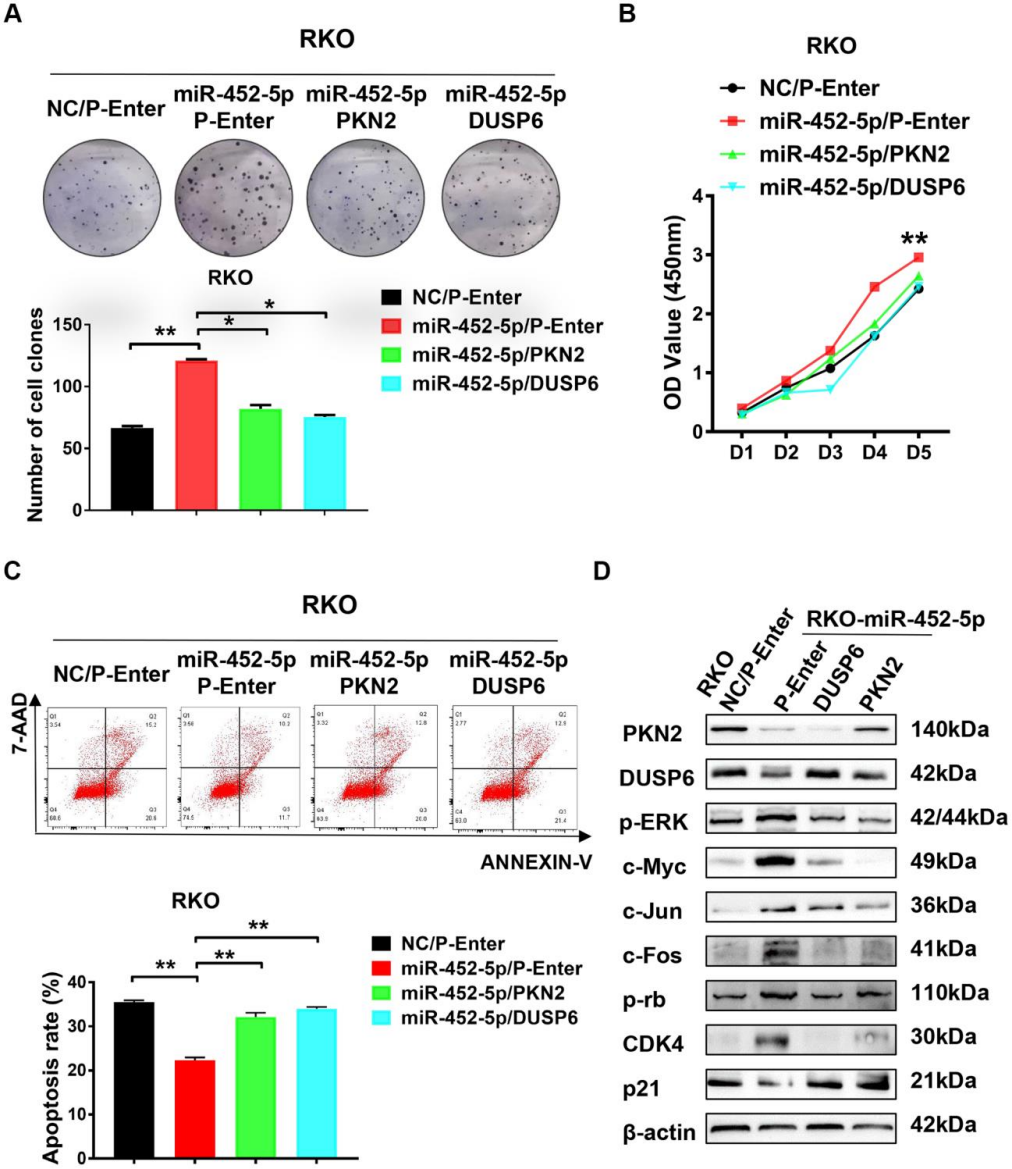
**PKN2/DUSP6 axis is involved in the miR-452-5p regulation of CRC cells biological behaviors.** (**A**) PKN2 and DUSP6 were verified to be involved in the regulation of miR-452-5p on colorectal cancer cell proliferation: panel cloning assay. (**B**) CCK8 assays showed that PKN2/DUSP6 reversed cell proliferation induced by miR-452-5p. (**C**) PKN2, DUSP6 were verified to be involved in the regulation of miR-452-5p on CRC cell apoptosis. (**D**) PKN2/DUSP6 axis was involved in the regulation of miR-452-5p on the important CRC signaling pathways.*p < 0.05, **p < 0.01, ***p < 0.001.

Colony formation assay was performed to verify whether PKN2/DUSP6 axis affected the effect of miR-452-5p on promoting CRC development. As a result, reversing the PKN2/DUSP6 axis restored the proliferation of cells induced by miR-452-5p (Figure 5A). Besides, RKO cells were used to perform CCK-8 assay, which also showed similar results that the PKN2/DUSP6 axis reversed the effect on promoting cell proliferation (Figure 5B). Next, we detected the effect of miR-452-5p-related apoptosis of 5-Fu-treated RKO cells. We found that such effect was rescued by restoring the PKN2/DUSP6 axis (Figure 5C). In line with the above findings, the PKN2/DUSP6 axis was suggested to play an important role in the development of CRC promoted by miR-452-5p. Furthermore, western blotting assay was performed to determine whether PKN2 or DUSP6 restored the activation of the ERK/MAPK pathway induced by miR-452-5p. According to our observation, the PKN2/DUSP6 axis played a vital role in suppressing the ERK/MAPK signaling proteins mediated by miR-452-5p, including p-ERK, c-Myc, c-Jun and c-Fos (Figure 5D and Supplementary Figure 3E). Also cell cycle and apoptosis signaling associated proteins mediated by miR-452-5p were restored by the PKN2/DUSP6 axis (Supplementary Figure 2C).

### MiR-452-5p activates the ERK/MAPK pathway and is transcriptionally regulated by c-Jun

**Figure 6:**
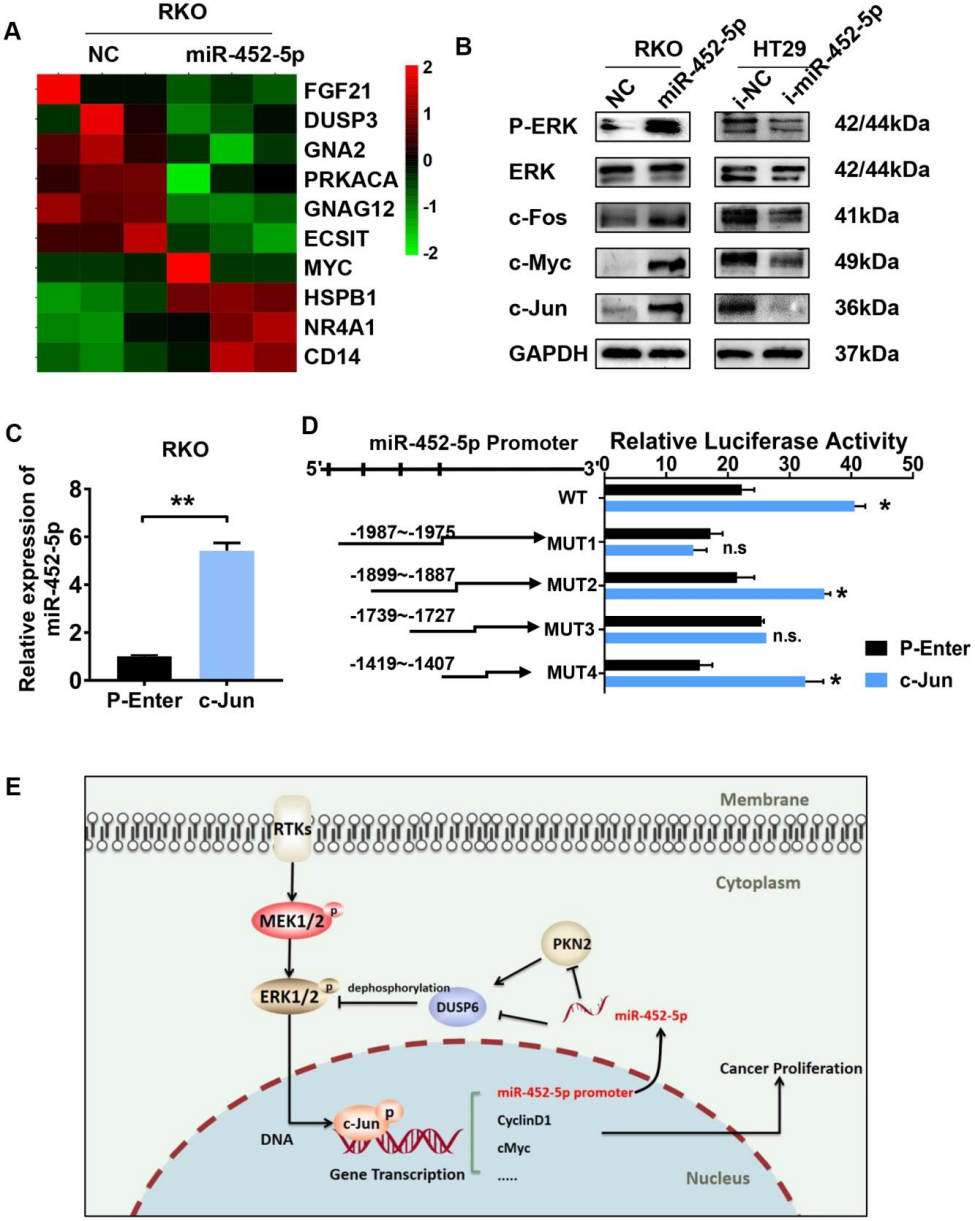
**C-jun regulates the expression of miR-452-5p by promoting its transcription.** (**A**) Heatmap analysis showed the MAPK signaling pathway genes as identified by sequencing. (**B**) Western blotting results of ERK/MAPK signaling pathway. (**C**) The expression of miR-452-5p after overexpression of c-Jun detected by qRT-PCR. (**D**) Luciferase reporter gene assay verified the direct transcriptional regulation effect of c-Jun on miR-452-5p. (**E**) A hypothetical model illustrating that miR-452-5p promotes the malignant behaviors of CRC through the miR-452-5p—PKN2/DUSP6—c-Jun positive feedback loop.*p < 0.05, **p < 0.01, ***p < 0.001, n.s. non-significant.

Previous sequencing results showed that the ERK/MAPK signaling pathway was enriched in RKO cells over-expressing miR-452-5p (Figure 6A). The ERK/MAPK signaling exerts an important part during the growth and differentiation of tumor cells. In this study, pathway-related proteins expression in CRC cells transfected with miR-452-5p inhibitors or mimic was measured for verification. The results suggested that, miR-452-5p activated the ERK/MAPK signaling by up-regulating p-ERK as well as the classical ERK/MAPK signaling pathway targets, such as c-Jun, c-Fos and c-Myc, while the expression level of ERK was not affected (Figure 6B and Supplementary Figure 3D). C-Jun, one of the canonical downstream activated by p-ERK, has been verified to be activated by the overexpression of miR-452-5p. Interestingly, we found that miR-452-5p expression increased in RKO cells over-expressing c-Jun (Figure 6C). To examine the effect of c-Jun on promoting miR-452-5p expression, we analyzed the miR-452-5p promoter region to predict the potential transcriptional factor binding sites of c-Jun using LASAGNA-Search. In addition, binding of c-Jun with miR-452-5p promoter region was determined by ChIP-PCR assay within the Flag tagged- c-Jun plasmids-transfected RKO cells. Afterwards, PCR was conducted to detect the miR-452-5p promoter fragments, so as to identify the candidate c-Jun binding sites. As shown, all the predicted sites were enriched by anti-Flag and anti-c-Jun antibodies relative to IgG control. These results suggested that, miR-452-5p was the c-Jun direct target in the context of CRC (Supplementary Figure 1E). In order to confirm whether c-Jun transcriptionally regulated miR-452-5p, we generated the promoter region of miR-452-5p (wild type) and four mutated predicted binding sites. According to our dual-luciferase assay, the promoter region of miR-452-5p (wild type) was found to significantly enhance the relative luciferase activity. Among the four mutated binding sites, positions −1899 ~ −1887 and −1419 ~ −1407 were found to significantly increase the relative luciferase activity, while positions −1987 ~ −1975 and −1739 ~ −1727 showed no significant effect (Figure 6D).

## DISCUSSION

Previous studies have illustrated that miR-452-5p may play dual roles in tumorigenesis and progression. MiR-452-5p is reported to promote tumorigenesis in hepatocellular carcinoma and CRC, and contributes to the docetaxel resistance of breast cancer cells [11, 13, 15]. On the other hand, it is also found to inhibit the progression of non-small cell lung cancer and prostate cancer [12, 14]. Nonetheless, further biological functions and molecular mechanisms of miR-452-5p in CRC remain undefined.

Firstly, in the current study, we found that miR-452-5p expression was up-regulated in CRC tissue samples relative to non-carcinoma intestinal epithelial tissue samples collected from TCGA database, which was verified in CRC tissues of our cohort. Moreover, overexpression of miR-452-5p was shown to promote malignant biological behaviors. MiR-452-5p exerted positive effects on cell cycle transition from G1 to S and promoted cell proliferation both *in vivo* and *in vitro*, but it negatively affected CRC cell apoptosis. In addition, when the miR-452-5p over-expressing cells were treated with 5-Fu, the IC50 value increased while the apoptosis rate decreased. This suggested that miR-452-5p reduced apoptosis and enhanced the chemoresistance of CRC to 5-Fu. These results indicated that miR-452-5p largely played a role of an oncogene in CRC. Numerous studies have identified that miR-452-5p is closely correlated with drug resistance [18–22]. MiR-452-5p is previously reported to contribute to the docetaxel resistance in breast cancer cells. Another research showed that Sunitinib, a receptor tyrosine kinase inhibitor (TKI) with multiple targets, suppressed RCC invasion and migration through down-regulating the expression of miR-452-5p. Besides, studies have shown that down-regulating miR-452-5p is related to the resistance of breast cancer cells to adriamycin. Such inconsistent functions of miR-452-5p are attributed to tissue specificity.

In this study, we performed RNA-sequencing (RNA-seq) analysis and subsequent bioinformatics analysis in RKO cells transfected with miR-452-5p and negative control. Numerous MAPK signaling pathway-related genes showed ectopic expression, indicating that this might be the downstream pathway of miR-452-5p. This hypothesis was then validated by Western blotting, which indicated that miR-452-5p aberrantly activated the ERK/MAPK signaling pathway and its downstream molecules, including p-ERK, c-Fos, c-Jun and c-Myc. The ERK/MAPK pathway is one of the most significant signaling pathways involved in cell proliferation, which is triggered by some crucial growth factors and genes [23, 24]. Moreover, accumulated studies have indicated that, the ERK/MAPK pathway is associated with the genesis, progression and metastasis of CRC [25, 26]. Researchers have contributed to constructing the novel inhibitors targeting the MAPK signaling pathway [27].

PKN2 and DUSP6 were identified as the candidate targets of miR-452-5p in this study. MiR-452-5p bound to the 3’UTR and thereby negatively regulated the expression of these genes. The DUSP family phosphatases represent the largest group of protein phosphatases that specifically inhibit the MAPK activity [28], which are reported to be dysregulated in CRC [29, 30]. DUSP6 is one of these protein phosphatases, which specifically prevents ERK phosphorylation. PKN2, acting as the Rho/Rac effector protein and the serine/threonine protein kinase related to PKC, has been confirmed to directly bind to the DUSP6 binding region to enhance the activity of DUSP6 in the dephosphorylation of Erk1/2. Thus, PKN2 functions as a tumor suppressor gene in the context of CRC [31].

Moreover, cell proliferation induced by miR-452-5p was reversed after restoring the expression of PKN2 and DUSP6, suggesting that the PKN2/DUSP6 axis at least partially diminished the malignant biological behaviors of CRC. In addition, the PKN2/DUSP6 axis might restrain the related ERK/MAPK pathway, apoptosis and cell cycle-related proteins. In brief, the PKN2/DUSP6 axis was closely involved in the ERK/MAPK signaling pathway, which regulated the malignant biological behaviors of CRC.

Finally, we found that c-Jun, a classical downstream transcription factor of the ERK/MAPK pathway, was the upstream of miR-452-5p by chromatin immunoprecipitation and dual-luciferase gene reporter assay. Previous studies have demonstrated that c-Jun plays an important role in the progression of CRC [32, 33] and it regulates miRNA and contributes to tumourigenesis in human cancers. [34, 35].

In summary, our study illustrates the promoting effect of miR-452-5p on CRC progression. MiR-452-5p binds to the 3’-UTR of PKN2 and DUSP6 directly, thus suppresses the PKN2/DUSP6 axis and activates the ERK/MAPK signaling pathway. C-Jun is a downstream factor of the ERK/MAPK pathway, which targets the promoter region of miR-452-5p. MiR-452-5p promotes CRC progression by forming the positive feedback loop of miR-452-5p—PKN2/DUSP6—c-Jun, (Figure 6E).

## MATERIALS AND METHODS

### Patients and clinical specimens

In this study, 87 CRC and corresponding adjacent non-carcinoma tissue samples were obtained from the Nanfang Hospital. The Ethics Committee of Nanfang Hospital Affiliated to Southern Medical University approved our study protocol. All patients provided the written informed consent for participation. Each patient in this clinical cohort had undergone standard surgery, and CRC was confirmed through postoperative pathology. The fresh tissue samples collected were then numbered and stored in liquid nitrogen for RNA extraction.

### Cells and treatments

Six types of human CRC cells, including HT29, RKO, LOVO, SW480, SW620 and HCT116, together with the colorectal mucosal FHC cells, were provided by the American Type Culture Collection (ATCC, USA) and preserved under liquid nitrogen condition. To be specific, the RKO, SW480, SW620, HCT116 and FHC cells were incubated within the Dulbecco’s modified Eagle medium (DMEM, high-sugar, Gibco, USA) that contained 10% fetal bovine serum (FBS; Gibco, USA), whereas HT29 and LOVO cells were incubated within the RPMI1640 medium (Gibco, USA) containing 10% FBS under 5% CO_2_ and 37° C conditions.

### RNA sequencing

The miR-452-5p mimics/negative control (NC) was transfected into RKO cells growing to 50% confluency, followed by 48 h culture under 5% CO_2_ and 37° C conditions. Further, we extracted the total cellular RNA and performed qPCR to confirm the transfection efficiency. Shanghai Biotechnology Corporation was responsible for RNA sequencing as well as data analysis.

### RNA extraction and real-time quantitative PCR (qPCR)

The total RNA was extracted via CRC tissues and cell lines using the Trizol reagent (Takara, USA). Thereafter, RNA purity and content obtained were measured using the NanoDrop2000 ultra-micro spectrophotometer. Then, the RNA reverse transcription system was prepared to synthesize cDNA following the instructions of the PrimeScript RT reagent Kit (Takara, USA). To synthesize the miRNA cDNA, we used the Mir-X^TM^ miRNA First-Strand Synthesis Kit (Takara, USA). Moreover, SYBR Premix Ex Taq (Takara, USA) was employed for qPCR in accordance with specific instructions. Data were analyzed according to the 2^–ΔΔCt^ approach, with GAPDH or U6 as the internal reference.

### Western blotting

For western blotting assay, the prepared protein samples were loaded into the SDS-PAGE gel, and then the PVDF membrane (Thermo, USA) was blocked with the 5% skim milk for 1 h after electrophoresis and transfer. Thereafter, the membrane was incubated using primary antibodies with appropriate bands at 4° C overnight. Later, the membrane was incubated using the suitable secondary antibody. Finally, Immobilon ECL (Millipore, USA) was utilized to visualize bands. The antibodies used in Western blotting in dilution 1:1000 are listed in Supplementary Table 2.

### Cell proliferation and colony formation assays

After transfection with appropriate plasmids for 48 h, cells were inoculated into the 96-well plate at a density of 1*10^3, followed by culture under 5% CO_2_ and 37° C conditions. For CCK8 assay, cells were incubated with 10 μl CCK8 (Dojindo, Japan) under the temperature of 37° C for 2 h. The Paradigm Detection Platform (Beckman, USA) was used to measure the values of optical density (OD) at the wavelength of 450 nm. Moreover, cell proliferation was detected in 5 days. With regard to colony formation analysis, cells were planted to the 6-well plate at a density of 200/well, followed by 14 days culture, 4% formaldehyde fixation and crystal violet staining.

### Cell cycle and apoptosis assays

Cells were harvested after 48 h transfection with appropriate plasmids, then fixed with 70% ethanol overnight under the temperature of 4° C and incubated with PI/RNase staining buffer (BD Biosciences, USA) to detect the cell cycle. To analyze the apoptosis, the transfected cells were treated with 10 μmol/L PBS or 5-Fu for 48 h. Then, those apoptotic cells were identified using the Annexin V-FITC/PI Apoptosis Detection Kit (BD Biosciences, USA) in accordance with the manufacturer’s protocols.

### Tumor xenograft nude mouse model

The 4-6-week-old nude mice were provided by Guangdong Experimental Animal Center and raised in the Laboratory Animal Centre of Nanfang Hospital under specific pathogen free (SPF) condition. Then, cells (at 5×10^6^) with stable over-expression of miR-452-5p or NC were injected to nude mouse right flank subcutaneously. Then, we monitored the animals regularly and measured the tumor volume at an interval of 5 days. At 30 days later, all mice were killed to harvest the xenograft tumors, which were then fixed with formalin and embedded in paraffin for further immunohistochemical analysis. The formula to calculate the tumor volume (mm^3^) was as follows, V= 1/2 × length × width^2^.

### Dual-luciferase gene reporter assay

The PKN2 (PKN2-WT) and DUSP6 (DUSP6-WT) wild-type 3′UTR, together with corresponding mutant plasmids, were constructed. Thereafter, we inoculated the RKO cells into the 24-well plate. Later, miR-452-5p mimics/NC and appropriate luciferase reporter plasmids were transfected into cells with 50% confluency and cultured for 48 h. Finally, the luciferase activity was detected using the Dual-Luciferase® Reporter (DLR™) Assay System (Promega, USA) in accordance with the protocols from manufacturer.

### Chromatin immunoprecipitation

The c-Jun-Flag-pEnter plasmids were transfected into RKO cells, followed by incubation for 48 h. Then, the chromatin immunoprecipitation assay was performed using the simpleChIP™ Enzymatic Chromatin IP Kit (CST, USA) following manufacturer protocols. In brief, sufficient cells were fixed with formaldehyde and a series of chromatin fragments (200-1000 bp) were obtained by the non-contact ultrasonic shredder. Further, the anti-c-Jun antibody (CST, USA), anti-Flag antibody (Sigma, USA), or IgG (NC) was used to perform immunoprecipitation. Later, the protein-DNA complexes were harvested and eluted for pure chromatin, and finally PCR was performed to confirm the c-jun-binding sites.

### Statistical methods

SPSS 19.0 was used for all statistical analyses. All experiments were carried out for thrice to collect data separately. A difference of P≤0.05 indicated statistical significance. Comparisons between two groups were analyzed by the independent sample Student’s t-test. The expression difference between matched normal and cancer tissues was detected by the paired t-test. Analysis of variance (ANOVA) was performed to determine Differences across at least three groups were determined by analysis of variance (ANOVA) and post-hoc analysis.

## Supplementary Material

Supplementary Figures

Supplementary Tables
