# Supplementary material for: MiR-452-5p promotes colorectal cancer progression by regulating an ERK/MAPK positive feedback loop
Source: Aging (Albany NY). 2021 Mar 3;13(5):7608–26. doi: 10.18632/aging.202657 (PMC7993669; doi:10.18632/aging.202657)
Supplement: Supplementary Figures [file aging-13-202657-s001.pdf]

SUPPLEMENTARY FIGURES

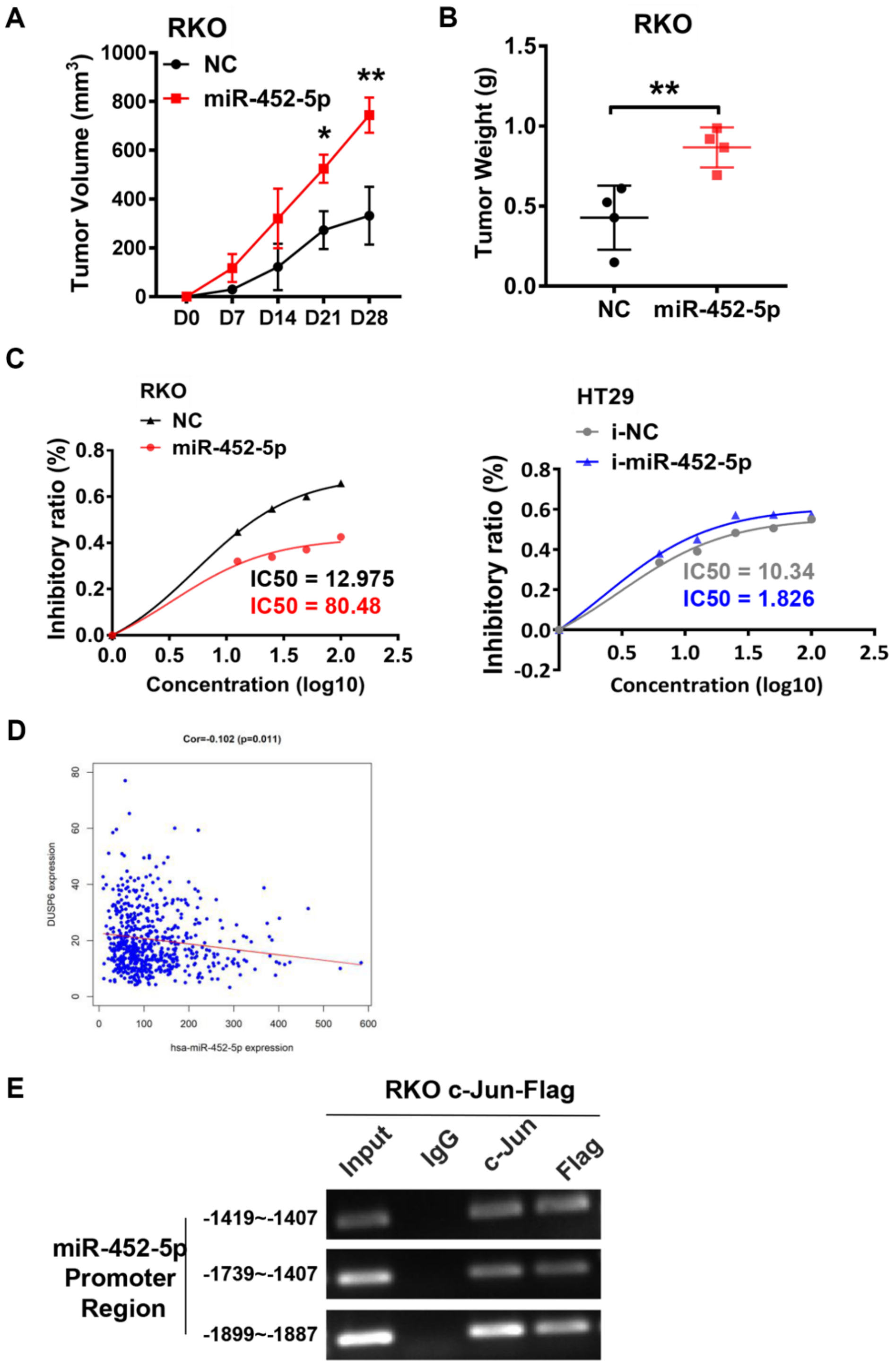

Supplementary Figure 1. (**A**) Subcutaneous tumor formation growth curve in nude mice. (**B**) Effect of overexpression of miR-452-5p on subcutaneous tumor formation weight. (**C**) Effects of overexpression and knockdown of miR-452-5p on chemosensitivity of CRC cells: IC50 test. (**D**) Spearman's correlation analysis showed a positive correlation between DUSP6 and miR-452-5p expression according to TCGA datasets. (**E**) Chip-PCR verified the c-jun-binding regions in miR-452-5p promoter.*p < 0.05, **p < 0.01, ***p < 0.001.

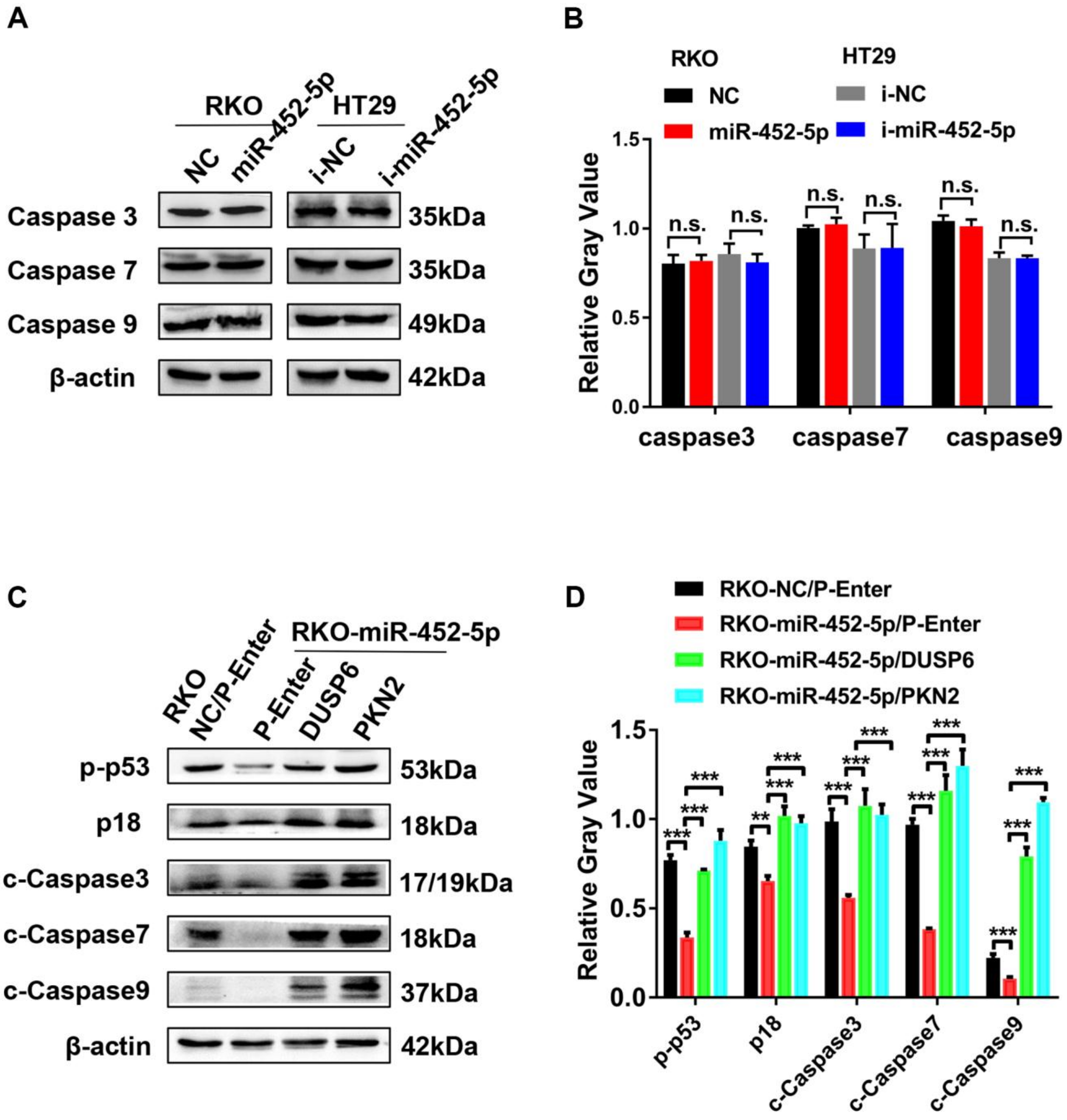

Supplementary Figure 2. (**A**) The expression of the full length caspase proteins detected by western blotting. (**B**) The average gray value analysis of the full length caspase proteins. (**C**) PKN2/DUSP6 axis was involved in the regulation of miR-452-5p on cell cycle and apoptosis signaling pathways. (**D**) The average gray value analysis of the protein expression of cell cycle and apoptosis signaling pathways after co-transfection with miR-452-5p mimics and PKN2, DUSP6 plasmids.*p < 0.05, **p < 0.01, ***p < 0.001, n.s. non-significant.

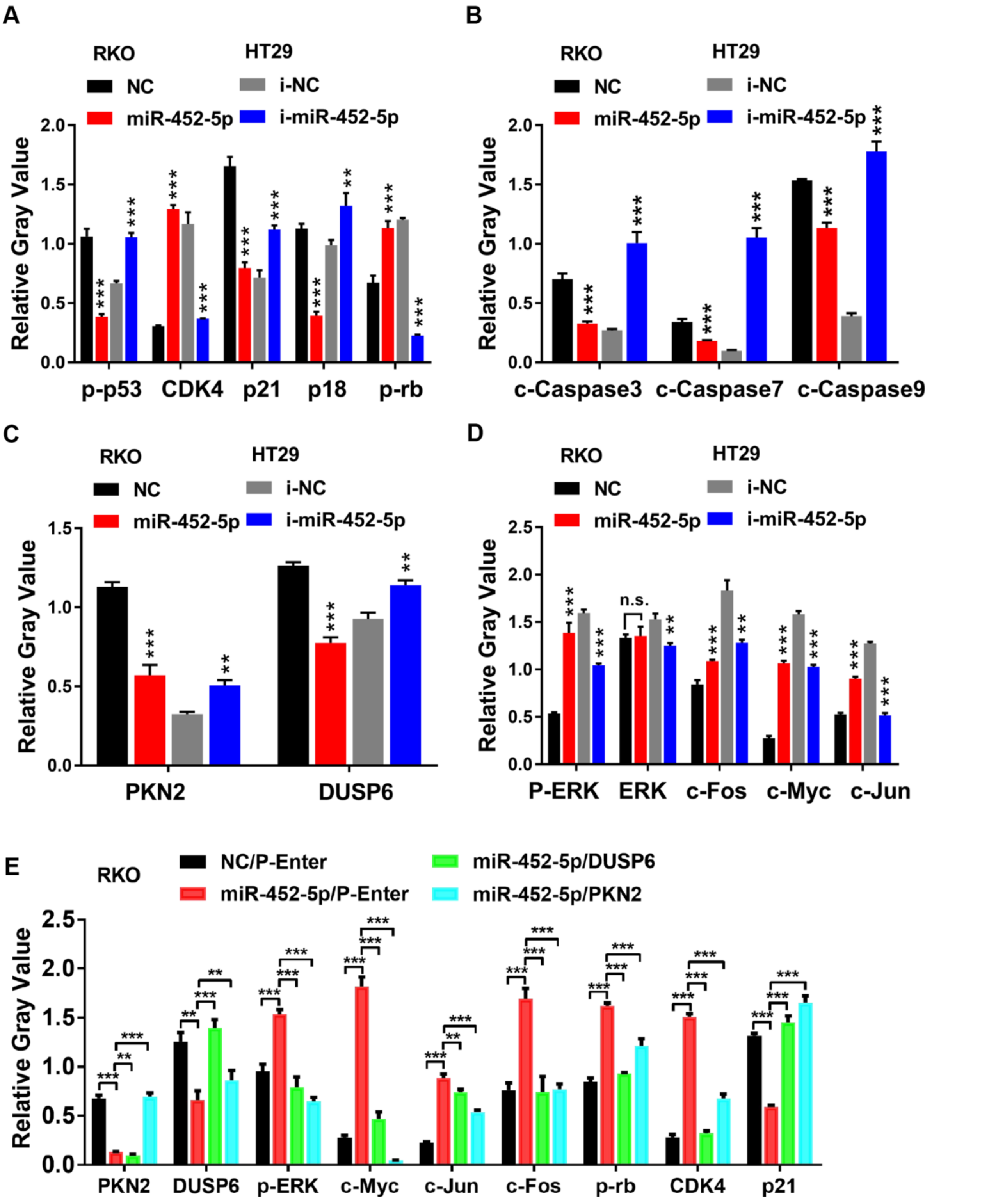

Supplementary Figure 3. The average gray values analysis of proteins (**A**) Cell cycle related proteins detected by western blotting. (**B**) The expression of apoptosis-related proteins detected by western blotting. (**C**) The expression of PKN2 and DUSP6 after overexpression and knockdown of miR-452-5p detected by western blotting. (**D**) Western blotting results of ERK/MAPK signaling pathway. *p < 0.05, **p < 0.01, ***p < 0.001, n.s. non-significant. (**E**) The average gray values analysis of protein expression of the important CRC signaling pathways after co-transfection with miR-452-5p mimics and PKN2, DUSP6 plasmids.*p < 0.05, **p < 0.01, ***p < 0.001.
