# Supplementary material for: MiR-452-5p promotes colorectal cancer progression by regulating an ERK/MAPK positive feedback loop
Source: Aging (Albany NY). 2021 Mar 3;13(5):7608–26. doi: 10.18632/aging.202657 (PMC7993669; doi:10.18632/aging.202657)
Supplement: Supplementary Tables [file aging-13-202657-s002.pdf]

SUPPLEMENTARY TABLES

Supplementary Table 1. Relationship between miR-452-5p expression and the clinicopathological features of CRC patients.

Variables	n	miR-452-5p expression		P value
		Low	High	
**Age (years)**				0.239
≥59yr	43	24	19	
<59yr	44	19	25	
**Gender**				0.143
Male	54	30	24	
Female	33	13	20	
**Tumor size**				0.74
>5cm	40	19	21	
≤5cm	47	24	23	
**Tumor location**				0.334
Rectal	43	19	24	
Colon	44	24	20	
**Histological grade**				0.546
Well/Moderate	71	34	37	
Poor/Undefined	16	9	7	
**pT status**				0.448
T1/T2	17	7	10	
T3/T4	70	36	34	
**pN status**				0.161
N0	46	26	20	
N1-N3	41	17	24	
**pM status**				**0.01**
M0	69	39	30	
M1	18	4	14	
**AJCC stage**				**0.042**
I/II	41	25	16	
III/IV	46	18	28	

According to the median expression of miR-452-5p, the CRC patients are divided into two groups: high expression group and low expression group.

Supplementary Table 2. List of antibodies used for western blotting.

Antibody	Catalog number	Company
Phospho-p53 (Ser15) (16G8)	9286	Cell Signaling Technology
CDK4 (D9G3E)	12790	Cell Signaling Technology
p21 Waf1/Cip1 (12D1)	2947	Cell Signaling Technology
p18 INK4C (DCS118)	2896	Cell Signaling Technology
Phospho-Rb (Ser795)	9301	Cell Signaling Technology
Cleaved Caspase-3 (Asp175) (5A1E)	9664	Cell Signaling Technology
Cleaved Caspase-7 (Asp198) (D6H1)	8438	Cell Signaling Technology
Cleaved Caspase-9 (Asp330) (E5Z7N)	52873	Cell Signaling Technology
Caspase-3 (D3R6Y)	14220	Cell Signaling Technology
Caspase-7 (D2Q3L)	12827	Cell Signaling Technology
Caspase-9 (C9)	9508	Cell Signaling Technology
PKN2 [E352]	ab32395	Abcam
DUSP6 [EPR129Y]	ab76310	Abcam
Phospho-p44/42 MAPK (Erk1/2) (Thr202/Tyr204) (D13.14.4E) XP	4370	Cell Signaling Technology
p44/42 MAPK (Erk1/2) (137F5)	4695	Cell Signaling Technology
c-Fos [EPR21930-238]	ab222699	Abcam
c-Myc [Y69]	ab32072	Abcam
c-Jun [E254]	ab32137	Abcam
GAPDH	60004-1-Ig	Proteintech
β-actin	66009-1-Ig	Proteintech
